# Posterior urethral polyps and review of literature

**DOI:** 10.4103/0970-1591.32080

**Published:** 2007

**Authors:** Prashant Jain, Hemanshi Shah, Sandesh V. Parelkar, S. S. Borwankar

**Affiliations:** Department of Pediatric Surgery, Seth G. S. Medical College and KEM Hospital, Mumbai, India

**Keywords:** Polyp, urethra, voiding cystourethrogram

## Abstract

Urethral polyp is a rare finding in young children. Fibroepithelial polyps of the urethra are usually diagnosed during the first decade of life. They present with obstruction, voiding dysfunction and hematuria. They can be associated with other congenital urinary tract anomalies. They are usually benign fibroepithelial lesions with no tendency to recur and are treated by surgical ablation, fulguration or laser therapy.

## INTRODUCTION

Urethral polyps are a rare cause of bladder outlet obstruction in the pediatric age group. They are benign fibroepithelial polyps usually diagnosed in the first decade and are not known to undergo malignant degeneration. Diagnosis is made by voiding cystourethogram and cystoscopy. We reported a rare case of multiple urethral polyp in an 11-month-old- male child.

## CASE REPORT

**Figure 1:**
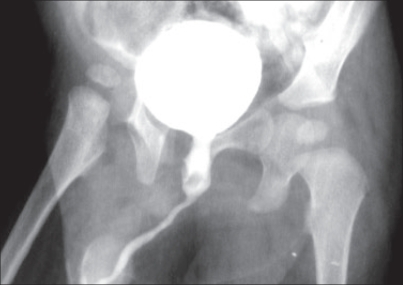
Voiding cystourethrogram showing dilated posterior urethra with two filling defects in the posterior urethra

An 11-month-old male child presented with complaints of excessive crying and straining during micturation. Patient was operated for low anorectal malformation during the neonatal period. There was no history of hematuria and urinary tract infection. Urine examination and renal chemistry were normal. Ultrasonography of the kidney and bladder was normal. Voiding cystourethrogram [Figure 1] demonstrated a dilated posterior urethra with two filling defects in the posterior urethra. Anterior urethra and bladder were normal.

**Figure 2:**
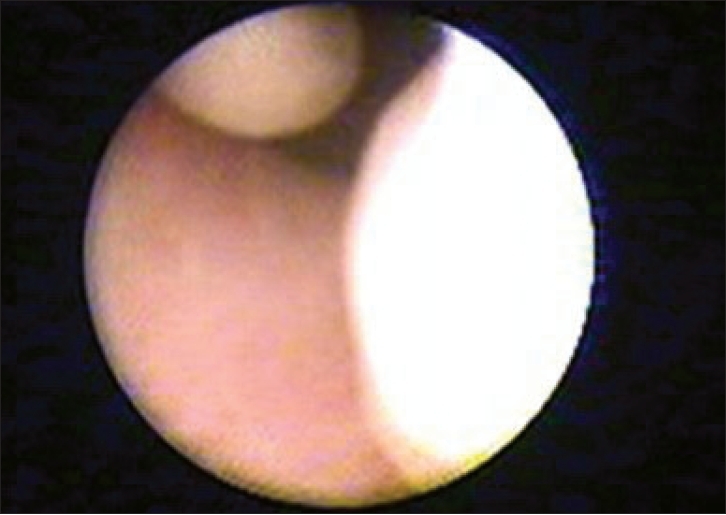
Cystoscopic view showing two urethral polyp in posterior urethra

A cystourethroscopy with 7.5Fr. cystoscope was done. Two pedunculated posterior urethral polyps proximal to the verumontanum at 11 o'clock and 2 o'clock position were seen [Figure 2]. Endoscopic fulguration of the base of both the polyps was done. Because of available small size working cystoscope polyp could not be retrieved and pushed into the bladder. Per urethral catheter was kept for two days. Patient is asymptomatic after two months of follow-up and repeat cystoscopy done showed no residual polyp.

## DISCUSSION

In 1763 Hunter first documented a urethral polyp that he described in an ox. Urethral polyps are rare in females and only three cases have been described in the literature.

Stephens[1] in 1963 reported five cases and Downs[2] in 1970 reviewed the literature of 30 cases of urethral polyp in males and added two more cases. David Kimcke and Dov Lask[3] reviewed the literature and reported a total of 50 cases. In a series of 1500 micturating cystourethrograms, Gatewood had one case of urethral polyp. Gary *et al*[4] reported five cases of urethral polyp and reviewed presentation of 48 cases of urethral polyp.

Foster *et al* in 1986 reported another five cases and including the cases of Zulian and Walker made a total count of around 60 cases reported in the literature. Castro *et al* in 1993[5] found 100 published cases in the literature and reported a series of 17 cases. A few other isolated cases have been reported in the literature.

Polyps arising from the verumontanum in children are congenital in origin. They are solid, epithelialized, fibrous tumors that arise on or in the vicinity of the verumontanum and lie along the floor of the posterior urethra.[1]

They have been speculated to present a developmental error in the invagination process of submucous glandular material of the inner zone of the prostate gland (Walsh *et al*, 1993). Downs (1970) has suggested that it is the abnormal protrusion of the urethral wall while Kuppuswami and Moors thought it to be changes in response to the maternal estrogen.

Average age of presentation as noted by Downs was 9.7 years.[2]

These polyps may present as acute urinary retention or intermittent obstruction due to prolapse into the bladder or urethral obstruction. On reviewing the presentation of 48 cases Kearney *et al*[4] found obstruction (48%) to be the most common presenting symptom followed by hematuria (27%) and retention (25%).

Although ultrasonography and voiding cystourethrogram are effective means of diagnosis, cystourethroscopy is confirmatory.

In the review of literature by Kimche and Lask,[3] of the 50 patients with average age of 8.25 years, dilatation of the upper collecting system was encountered in 10 patients and reflux was seen in four patients. Additionally, bladder diverticulum was present in four patients. Although multiple polyps are more common in adults no reports of multiple polyps in pediatric patients were found in the literature. This is probably the first reported case of multiple polyps in a pediatric patient. Our patient did not have any upper tract anomaly. On reviewing the literature we could not find any association of posterior urethral polyp with anorectal malformation as was in our case.

Polyp can be accessed by suprapubic approach or transurethral excision. In the 50 cases described in the literature 16 polyps were excised by transurethral approach while the remaining 34 were removed transvesically. The latter approach seems to be easier in treating a young patient with large polyp.[3] De Wolf and Fraley reported transurethral excision via perineal urethrostomy in a three-week-old neonate.

De Castro[5] managed 17 cases of posterior urethral polyps endoscopically without complications or relapses. Open surgery should be used only when transurethral resection is not possible.
